# Combined Cytomegalovirus Retinitis and Vitreous Hemorrhage in an Immunocompromised Patient

**DOI:** 10.1155/crop/5510040

**Published:** 2025-05-23

**Authors:** Abdulrahman Y. Alhoumaily, Abdulsalam M. Dheyab

**Affiliations:** College of Medicine, King Saud University, Riyadh, Saudi Arabia

**Keywords:** cytomegalovirus retinitis, immunocompromised, occlusive vasculitis, vitreous hemorrhage

## Abstract

**Purpose: **This study is aimed at describing a case of combined CMV retinitis and vitreous hemorrhage in an immunocompromised patient.

**Observations:** A 38-year-old male who is known to have HIV presented to our emergency department complaining of decreasing vision in his left eye. Vitreous hemorrhage and preretinal hemorrhage were observed upon examination and were thought to be caused by CMV retinitis. After initial treatment and worsening condition upon follow-up, a diagnosis of combined CMV retinitis and vasculitis was considered and was treated accordingly.

**Conclusions:** The presence of vitreous hemorrhage in immunocompromised patients should not be attributed to just an ischemic vasculitis alone, and the possibility of concurrent infectious retinitis should be looked for carefully to avoid delay in treatment.

## 1. Introduction

Cytomegalovirus is a double stranded DNA virus attributed to the Herpesviridae group of viruses. Usually, it is asymptomatic in immunocompetent individuals, but in a low percentage, it can cause mononucleosis [1]. Other manifestations, which are increased in immunosuppressed states, include hepatitis, esophagitis, pneumonitis, and transverse myelitis [2]. Ophthalmic manifestations include CMV iridocyclitis, CMV endotheliitis, CMV retinitis, and CMV retinal vasculitis [3]. CMV iridocyclitis and CMV endotheliitis are typically linked to immunocompetent individuals, while CMV retinitis and CMV retinal vasculitis are more likely to be associated with immunocompromised patients. Vitreous hemorrhage has been described in a minority of cases of CMV retinitis, mostly secondary to retinal ischemia and occlusive vasculitis.

The incidence of the various manifestations caused by this virus is highly dependent on patient immune status. In regard to ocular involvement, posterior segment involvement, that is retinitis and ischemic vasculitis, is correlated with lower CD4+ counts in comparison to anterior segment involvement. In these states, CD4+ counts are usually < 200 cells/microliter [4]. Systemic anti-CMV treatment is usually needed in CMV retinitis and vasculitis, while in anterior segment involvement, systemic, topical, and local antiviral medications are tried, with no consensus about the best modality of treatment [5].

## 2. Case Description

**Figure 1:**
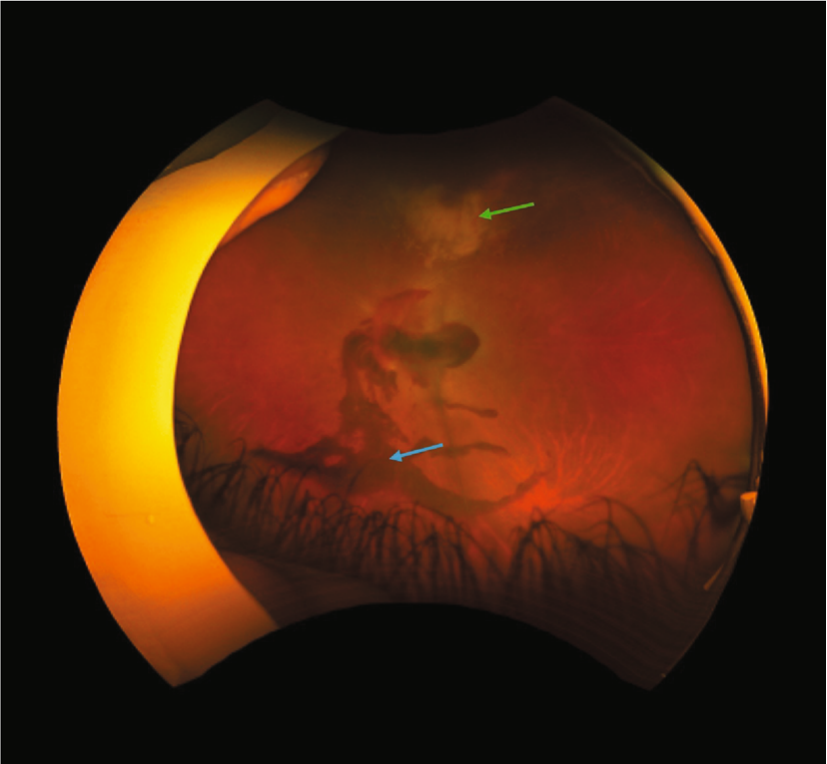
Left fundus photo showing areas of retinitis (green arrow), vitreous hemorrhage (blue arrow), and preretinal hemorrhage over the disc and macula.

**Figure 2:**
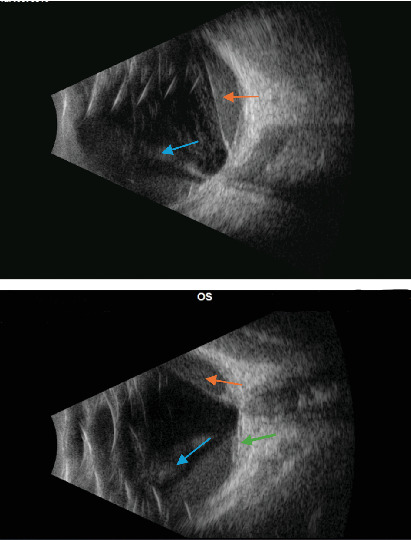
B-scan of the left eye demonstrating vitreous hemorrhage (blue arrow), preretinal hemorrhage (orange arrow), and premacular thickening (green arrow).

**Figure 3:**
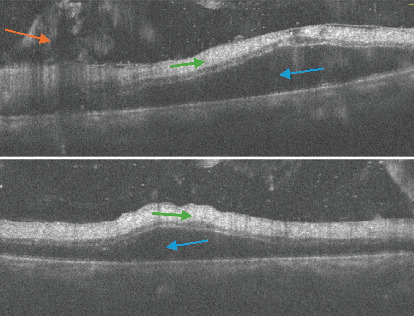
OCT of the left eye. Cut is at the macula and is showing preretinal hemorrhage (orange arrow). Subretinal fluid (blue arrow), hyperreflectivity of retinal layers, and loss of retinal layer stratification (green arrow).

Our patient is a 38-year-old male, a known case of HIV, for which he is currently on emtricitabine/tenofovir, alafenamide, and dolutegravir treatment for the past 2 months. The patient's past ophthalmic history is unremarkable. He presented to our emergency department complaining of decreasing vision in the left eye. His UCVA in the left eye was counting fingers (CFs) and 20/20 in the right eye. He reported no flashes, floaters, or any history of trauma. Slit lamp examination of the left eye showed mutton-fat keratic precipitates as well as an anterior chamber reaction. Fundus examination showed vitritis associated with vitreous hemorrhage and areas of retinitis (Figures 1 and 2). An area of preretinal hemorrhage over the disk and part of the macula was detected, but proper visualization of the disk and retina was not possible because of the vitreous hemorrhage. OCT of the left eye showed vitreous opacities, preretinal hyperreflective material suggestive of preretinal hemorrhage, subretinal fluid, hyperreflectivity of inner retinal layers, and loss of retinal layer stratification, suggestive of severe retinal ischemia (Figure 3).

**Figure 4:**
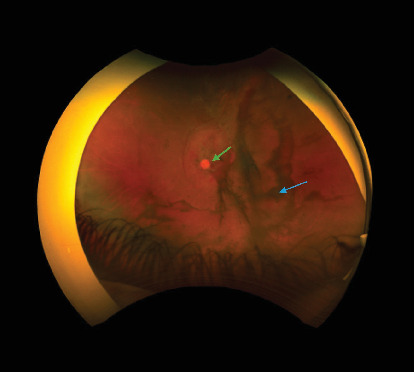
Left fundus photo showing persistent vitreous hemorrhage (blue arrow) and preretinal hemorrhage over the disc (green arrow).

Because of the immune status of the patient and the presence of hemorrhagic retinitis, the diagnosis of cytomegalovirus retinitis was considered and speculated. Although vitreous and preretinal hemorrhage was not explained by this preliminary diagnosis, our initial thought was that it may be due to posterior vitreous detachment secondary to the vitritis or due to the inflammation. The patient was treated as a case of CMV retinitis and was started on valganciclovir 900 mg BID and a series of intravitreal injections of ganciclovir. Upon follow-up, retinitis seemed to improve but the preretinal hemorrhage as well as the hemorrhage over the disk seemed to persist (Figure 4). One month later, slit lamp examination showed neovascularization on the iris (NVI). Fluorescein angiography revealed ischemic retina, and the patient was planned to have panretinal photocoagulation as well as intravitreal injection of Avastin.

**Figure 5:**
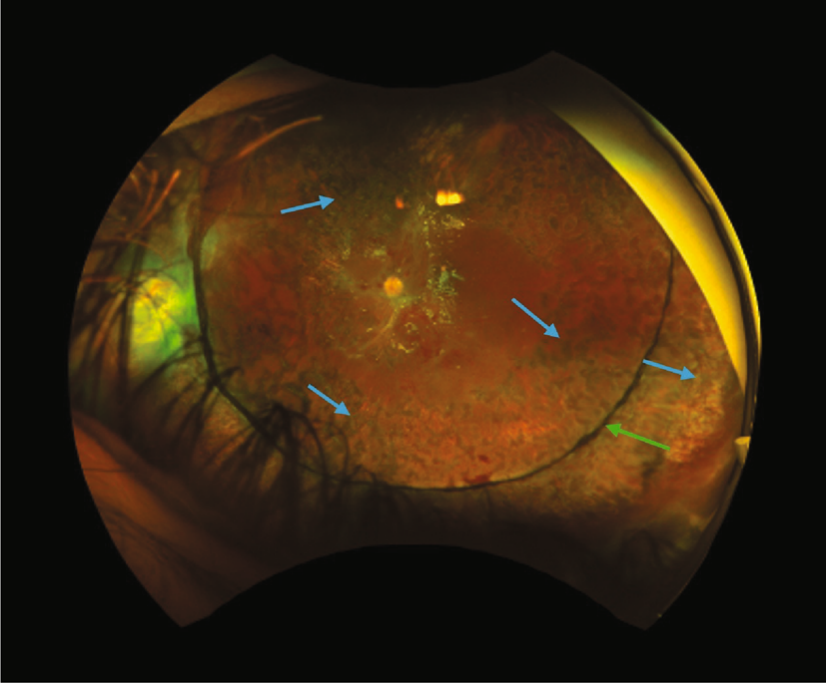
Postoperative fundus photo showing silicon-oil filled eye (green arrow) with panretinal photocoagulation scars (blue arrows) and pale optic disc.

On the next follow-up appointment, the patient presented with rapidly decreasing vision and increasing vitreous hemorrhage. Pars plana vitrectomy was planned and done. After vitrectomy and aspiration of the preretinal hemorrhage, a fibrovascular membrane over the disk and macula was found. Peeling of the membrane, augmentation with laser photocoagulation, and silicone oil injection were done (Figure 5).

These intraoperative findings, along with the angiography findings, suggested ischemic retinitis and vasculitis, most likely secondary to the CMV infection. During the postoperative follow-up period, the patient demonstrated improving vision, but the macula appeared to be atrophic, most likely due to the ischemia and vasculitis. At final follow up, the patient had a UCVA of 20/200, and the elements of retinitis and vasculitis resolved.

## 3. Discussion

CMV retinitis is a common ocular opportunistic infection in immunocompromised patients. It is usually associated with retinal vascular changes, and retinitis typically starts and distributes along the retinal blood vessels. It is suggested that the retinal vascular changes are secondary to the CMV infection, leading to retinal ischemia with possible retinal neovascularization, vitreous hemorrhage, or traction retinal detachment [6]. Immunohistochemical studies detect CMV proteins in the retinal vascular endothelium adjacent to the areas of retinal involvement, with the vascular endothelium being proposed as the primary site of CMV infection in the retina. In HIV patients, the exact role of HIV vasculopathy in the development of CMV retinitis is still controversial [7]. Despite the common association with retinal vasculitis and retinal ischemia, CMV retinitis rarely presents concurrently with vitreous hemorrhage.

Ch'ng et al. reported a case of CMV retinitis presented as vitreous hemorrhage in a patient with T-cell prolymphocytic leukemia treated with a course of alemtuzumab [8]. However, most other case reports reported vitreous hemorrhage in CMV retinitis after treatment or healing of CMV retinitis, but not as a presenting sign with active CMV retinitis [9–12].

Some other case reports attributed vitreous hemorrhage in HIV-infected patients to immune recovery after starting HAART therapy, and the authors thought that the retinal neovascularization and vitreous hemorrhage were caused by retinal ischemia and were induced by an enhanced immune response to CMV in the retina after partial immunologic restoration from HAART therapy [13–15]. In our case, being an HIV patient with hemorrhagic retinitis suggestive of CMV retinitis and on HAART therapy, the role of HIV vasculopathy, CMV vasculopathy, and HAART-induced immune recovery vasculopathy cannot be ruled out, and they could all contribute to the development of this unusual presentation of combined vitreous hemorrhage with active CMV retinitis.

## 4. Conclusion

The presence of vitreous hemorrhage in immunocompromised patients should not be attributed to just an ischemic vasculitis alone, and the possibility of concurrent infectious retinitis should be looked for carefully to avoid delay in treatment, with the possibility of disastrous visual outcome.

## Data Availability

The data that support the findings of this study are available on request from the corresponding author. The data are not publicly available due to privacy or ethical restrictions.
